# Stresses, Aging, and Age-Related Disorders

**DOI:** 10.1155/2014/320564

**Published:** 2014-12-30

**Authors:** Xiaotao Li, Robb E. Moses, Jianping Jin, Weiguo Cao, Carlos Caulin

**Affiliations:** ^1^Department of Molecular and Cellular Biology, Baylor College of Medicine, Houston, TX 77030, USA; ^2^Key Laboratory of Brain Functional Genomics, Ministry of Education and Shanghai Key Laboratory of Regulatory Biology, Institute of Biomedical Sciences, School of Life Sciences, East China Normal University, Shanghai 200241, China; ^3^Department of Biochemistry and Molecular Biology, Medical School, The University of Texas Health Science Center at Houston, Houston, TX 77030, USA; ^4^Department of Genetics and Biochemistry, Clemson University, Clemson, SC 29634, USA; ^5^Department of Head and Neck Surgery-Research, UT MD Anderson Cancer Center, Houston, TX 77030, USA

This special issue focuses on the topics of aging and its related disorders and stress. Stress, defined as a pressure or tension on a subject, can be in any of many forms. As related to aging, two general areas of stress have been entertained as contributing to the process: genome damage and metabolic deterioration. While neither area satisfactorily explains the aging process, each supplies useful data. Indeed, the metrics used to monitor the aging process are unsatisfactory; a simple concise definition of aging has yet to be established. Moreover, the concept of genetic control of aging presents a dilemma; if there is none, why does a dog live only seven or eight years with a metabolism not radically different from a human? Yet if genetic programming controls the process exclusively, what are the master genes? We are left to hypothesize on bases for aging involving both environmental and genetic elements [1].

There are several keystone observations relating to aging which give opportunity for study [1]. First, the observation that calorie restriction increases life span in rodents [2] has been repeated for over seventy years and is robust [1, 3]. While studies on primates are inconclusive, in rodents,* S. cerevisiae, C. elegans*, and* Drosophila* results appear unambiguous. Secondary to this, in yeast and worm, later in the fly, a set of genes regulated by calorie deprivation was identified. These observations led to the identification of the SIRT genes in higher organisms, a highly conserved family involved in regulation of cellular NAD+ levels and energy expenditure. It has been possible to construct long-lived mutants for the worm, establishing genetic components to longevity [4].

A second area of seminal observations relates to oxidative damage to the cell. These studies relied on the recognition of accumulation of genome errors (mutations) and demonstration of degraded protein synthesis in aged systems compared to young systems. This has led to the school of thought that free radicals such as reactive oxygen species (ROS) can produce accumulated damage and lead to cellular senescence [5]. This has led to focus on the state and function of mitochondria and relation to energy metabolism in the face of free radical insult. That said, there is no evidence supporting rescue of senescence by use of reducing agents or antioxidants, despite extensive trials. However, schemes linking calorie restriction and mitochondrial function are plausible.

It is in this line of thought that we have included an emphasis on mitochondrial function and oxidative stress in this special issue. Hypotheses remain at the interface of reduced calorie consumption and its metabolic signaling and the response of a handful of genes which can prolong lifespan.

*Xiaotao Li* *Xiaotao Li* *Robb E. Moses* *Robb E. Moses* *Jianping Jin* *Jianping Jin* *Weiguo Cao* *Weiguo Cao* *Carlos Caulin* *Carlos Caulin*
